# A specific cognitive behavioral group therapy program for stimulant use disorder

**DOI:** 10.3389/fpsyt.2022.1031067

**Published:** 2022-11-30

**Authors:** Emily Karsinti, Florence Vorspan, Norman Therribout, Romain Icick, Vanessa Bloch, Maeva Fortias, Kristel Piani, Lucia Romo

**Affiliations:** ^1^Clinique Psychanalyse Développement, Unités de Formation et de Recherche Sciences Psychologiques et Sciences de l’Education, Université Paris Nanterre, Nanterre, France; ^2^Hôpital Fernand Widal, Département Universitaire de Psychiatrie et d’Addictologie, Paris, France; ^3^INSERM U1144 Variabilité de Réponse aux Psychotropes, Paris, France; ^4^FHU Network of Research in Substance Use Disorders, Paris, France; ^5^Université Sorbonne Paris Cité, Paris, France; ^6^Hôpital Raymond-Poincaré, Garches, France

**Keywords:** group therapy, substance use disorders, cocaine (PubChem CID: 11302220), Cognitive Behavioral Therapy (CBT), craving

## Abstract

**Introduction:**

Stimulant use is an important health issue. In the US in 2018, 2.8% of males and 1.5% of females older than 18 had used cocaine in the preceding 12 months.

**Objective:**

To intervene in a specific targeted group of Stimulant Use Disorder (SUD) patients according to CBT and relapse prevention theories, and to determine the program’s feasibility and attendance.

**Method:**

Stimulant Use Disorder patients in addiction care were evaluated for addictive, psychological and psychiatric dimensions at baseline and conclusion in a 9-session CBT group program with several themes: define SUD, enhance motivation, involve close companions, cope with craving, decline a proposal, solve problems, invite expert patients, invest time and money, and review content.

**Results:**

In total, 41 patients attended at least one session. They were mainly poly dependent, primarily cocaine users. Sixty percent of the population also suffered from another psychiatric comorbidity. Median attendance for participants was 7/9 sessions.

**Conclusion:**

A specific targeted CBT group for stimulant dependent highly comorbid patients is feasible. These findings suggest that peers should be included in addiction care services.

## Introduction

### Prevalence

Stimulant use is an important health issue. In the US in 2018, 2.8% of males and 1.5% of females older than 18 had used cocaine in the preceding 12 months (1), a number close to that in Europe in 2019, where 2.1% of 15 to 34 year olds had taken cocaine in the past 12 months, 1.4% amphetamines, and 1.9% MDMA (3,4-Methylenedioxymethamphetamine). In France these numbers are even higher (3.2, 0.6, and 1.3%, respectively) (2). The use of New Synthetic drugs, including cathinones and the non-stimulant synthetic cannabinoids is estimated at 1.1% among this same population in Europe. For the methamphetamine, some countries include it in their amphetamine use data and the prevalence rate seems to be highly variable, between 330 and 34,600 users at risk per country.

Stimulants use, including cocaine, has many consequences, including somatic (infarctus, pulmonary insufficiency, stroke…) (3, 4), psychiatric (a higher incidence of anxiety disorders or induced psychotic symptoms) (5, 6), and social consequences. Moreover, in the United States from 2010 to 2014, on average 7,500 of the 40,000 overdoses per year involved stimulants (cocaine or methamphetamine), and overdoses per year with these substances are rising (7).

### Specificities of stimulant users

Stimulant users attending care programs represent a specific population in many ways. Indeed, they are at high risk of experiencing delusional thinking (30% of cocaine-dependent patients) and unusual social or sexual behavior (65%) (5). They show a very strong association with childhood trauma. A previous study found that 62% of cocaine users had experienced such trauma (8). Furthermore, cocaine users are largely poly users. In another study they presented a median of three lifetime DSM IV dependence to other substances than cocaine (9). These substances were mostly “downers,” substances sharing sedative properties (alcohol, benzodiazepine, and cannabis), so patients are likely to use them to prevent coming-off effects.

In France, among stimulant users, cocaine users who entered treatment centers are mostly men (80%) and on average started cocaine at 24 years old and entered the center at 33 years old, meaning that there is a great delay between first use and treatment access (10). The management of stimulant users is characterized by several barriers to treatment. In substance abuse clinics, 34% quit the process within two months, and cocaine-related issues increased the risk of early drop-out (11). Of methamphetamine users, only 23% of outpatients remained in treatment after 180 days (12).

Moreover, significant neurocognitive impairment has been shown among cocaine users (13). A meta-analysis suggests that impulsivity is a core process underlying addictive disorders (14). A study comparing cocaine users to healthy controls found that cocaine users have elevated scores on trait impulsivity and have significantly poorer performance on inhibition and perseveration (15). Furthermore, dependent cocaine users display broad cognitive impairments in the domains of attention, working memory, declarative memory, and executive functions compared to recreational users or non-cocaine users (16).

### Treatment

Because there are no validated pharmacotherapies for stimulant treatment, psychotherapy seems to be an important part of the treatment. Cochrane Library published a meta-analysis in 2016 of 52 controlled randomized trials of psychotherapies for stimulant treatments, finding that all individual interventions diminished drop-out rates and enhanced abstinence (17). Another meta-analysis in 2018 of studies of cocaine and amphetamine users found that the combination of two different psychosocial interventions, contingency management and community reinforcement, was the most efficacious and accepted treatment in the short and long term (18). More recently, a systematic review published in 2020 states that no pharmaceutical intervention has proven its efficacy and the most promising psychological intervention was Contingency Management (CM). This therapy seems to have a short-term efficacy on abstinence. Moreover, the combination of CM and Cognitive Behavioral Therapy might be the most efficient therapy with a higher rate of abstinence, a lower drop out and probably more long-term effect. About CBT alone, the authors conclude that more research is needed to ensure its efficacy, particularly on abstinence (19).

The French High Health Authority recommends individual psychotherapy, such as Cognitive Behavioral Therapy (CBT), for cocaine dependence and states that groups could provide an interesting complement (20). They suggest a number of themes that could be discussed: Managing craving, enhancing motivation, gaining competences to resist solicitations, recognizing high-risk situations, generalizing strategies to face the desires to consume, and solving urgent problems that could pose the risk of using cocaine. Furthermore, Marlatt and Donovan suggest that Relapse Prevention for stimulant use should include an initial evaluation with common objectives, then a large part of the therapy should focus on cravings (trigger identification, exposition, refusal to use, alternative strategies, etc.) (21). Stimulant use can cause neuropsychological impairment that must be taken into account before engaging in any therapy, and it is preferable to postpone the relapse prevention program after a neurocognitive training to enhance the efficacy (22).

Because of the cost of the individual setting and the contribution of peer groups, there have been several studies of group therapies for stimulant users. The Matrix Program combines individual and group sessions (relapse prevention, 12-step, family, and social support groups) (23). Furthermore, Tzilos et al. developed a contingency model for cocaine users in methadone-maintained treatment. Among them, 26% never came to any session and 62% were non-completers (completers were defined as patients who came to at least six consecutive sessions with cocaine-negative urine samples) (24). A Spanish team developed a combined CBT and motivational open group 12-session program for cocaine users that demonstrated a very high retention rate (84%) (25). A study comparing CBT and Mindfulness Treatment (MT) open groups among drug users (alcohol and/or cocaine) showed high drop-out and similar drug reduction in both groups (26). In this study, the CBT program was implemented according to the National Institute for Drug Abuse guidelines (27). They suggest several topics to work with the patient: Coping with craving, shoring up motivation and commitment to stop, refusal skills/assertiveness, seemingly irrelevant decisions, coping plans, problem solving, case management, and HIV or other infectious risk reduction.

Several studies of cocaine treatment include avoidance and reinforcement components, but a large study illustrated the ineffectiveness of punitive approaches and highlighted the potential of improving goal-directed behavior and employing more desirable habits to replace drug-taking habits, such as CBT approaches (28). The third wave of CBT approaches, specifically Mindfulness Based Interventions, seems to have a significant effect on craving and substance misuse, so this approach could constitute a useful therapy for addiction treatment (29).

Therefore, the aim of this study was to intervene in a specific, targeted group for stimulant dependent patients. Few sessions were designed to take into consideration the impulsivity and lack of persistence of these patients in order to enhance attendance. The conceptual framework was CBT and relapse prevention theories. The secondary objective was to observe the feasibility, acceptability, and attendance in this group.

## Materials and methods

### Participants

Participants were recruited from the clinical outpatient department of a university hospital in Paris (France). Potential participants were identified by their treating psychiatrist or psychologist. The inclusion criteria were: (1) regularly followed French-speaking patients, (2) met diagnostic criteria of Substance Use Disorder (SUD) according to the Diagnostic and Statistical Manual for Mental Disorder 5 (30) for any stimulants (cocaine, crack, amphetamines, methamphetamine, cathinones), (3) wanting to stop or diminish their consumption, and (4) without acute psychiatric symptoms preventing group participation such as current delusion, hallucinations, mood instability, or suicidal ideation. No psychiatric diagnosis was excluded. All participants joined the same therapy program as an add-on to their usual outpatient psychiatric and addiction medicine care.

### Ethics

The study was conducted according to the Declaration of Helsinki and the French legislation on biomedical research in human subjects (Loi Jardé 2014), as well as the ethical guidelines of our hospital for the analysis of data already collected during routine care (Authorization 2017–013 given on 19 January 2017 by the CNIL, the Commission Nationale Informatique et Liberté, or French National Board for Information Systems and Freedom). Verbal consent to participation and research application of the data was obtained from all participants after information. Furthermore, specific information and consent was obtained for relatives’ participation.

### Assessment at entry

Eligible participants were invited to an initial visit to discuss participation and receive information about the group therapy program, rules, and assessments in the month before the session started.

#### Clinical evaluation

Socio-demographic data was collected in a semi-directed interview with the therapist, as well as substance use histories (substance use disorders, age at onset, and routes of administration). Psychiatric diagnoses and actual psychotropic treatment were recorded from the medical record. Attendance was recorded as the number of sessions attended by each individual and participant subjective feed-back was recorded during the last session (no. 9).

#### Hospital anxiety and depression

Actual anxiety and depression were assessed using the HAD (Hospital Anxiety and Depression) screening questionnaire, a 14-item self-rated questionnaire that evaluates anxiety and depression during the past week (31). A Canadian study showed that this measure seems to have a good level of reliability with Cronbach alpha around 0.8 and confirm the two factors measure with anxiety and depression subscales. Their results were similar among the general population and multimorbidity patients (32).

#### Timeline followback

Stimulant frequency and intensity of use were evaluated with the TLFB (TimeLine Followback) questionnaire. This tool is a calendar (initially developed for alcohol consumption) in which patients note when they use a drug and how much (33). The tool has proven its reliability using test-retest comparison for several substances. Also, the TLFB has demonstrated its validity, being highly correlated with the Addiction Severity Index and discrimination with high correlations with urine sample analysis (34).

#### Brief situational confidence questionnaire

Self-confidence was recorded with the BSCQ (Brief Situational Confidence Questionnaire) questionnaire, a state-dependent measure that assesses self-confidence to resist the urge to use a drug in several situations with 8 items in a Visual Analogic Scale (35). A study among incarcerated youth highlights a good test-rest stability (Pearson’s r around 0.60) and internal consistency (Cronbach’s alpha around 0.85) (36).

#### Obsessive compulsive craving scale

Craving was assessed using the OCCS questionnaire (Obsessive Compulsive Craving Scale), which is a 14-item questionnaire with a total score and two subscales: obsession and compulsion during the last 2 weeks (37). This same study shows a Cronbach’s alpha of 0.93. It also highlights a high correlation with the Visual Analog Scale (Pearson’s *r* = 0.641).

#### University of rhode island change assessment

The motivation to change was evaluated with the URICA (University of Rhode Island Change Assessment), a 32-item self-rated questionnaire to evaluate change motivation on four subscales (pre-contemplation, contemplation, action, and maintenance), with a total score calculated by adding the scores for contemplation, action, and maintenance and subtracting the pre-contemplation score (38). Each subscale has good internal consistency with Cronbach’s alpha ranging from 0.81 to 0.88 (39).

### Design of the therapeutic intervention

This closed group consisted of nine sessions with two therapists, each of 1.5 h duration. The authors did design this group intervention according to classical Relapse Prevention themes (21, 40, 41). The synthetic themes of each session are presented in Table 1. Sessions included several themes such as: a common definition of TUS according to DSM 5 (30), introduction to motivational interviewing and Prochaska and Di Clemente’s theory (42); introduction to assertiveness principle (43), solving problem strategies (44) and relaxation (19, 45).

**TABLE 1:** Sessions and themes.

Session	Theme	Content
1	**Define substance use disorder:** ● Patient’s criteria ● DSM 5	The patients fill the questionnaires, then the therapists remind them the rules of the group (listening, non-judgment attitude and confidentiality, attendance, punctuality, the need to come sober) and give patients a booklet containing session titles and empty spaces to be completed during the sessions. The aim of this session is to create a link among patients and to define Substance Use Disorder (SUD) according to the Diagnostical and Statistical Manual 5 definition (DSM 5). Patients perform a photolanguage exercise. They have to pick an image that defines their stimulant dependence and explain why. From this material, patients define their own SUD criteria, eventually completed by the therapist. Finally, harm reduction strategies are proposed.
2	**Enhance motivation:** ● Present stages of change ● Decisional balance	This session focuses on change, following Prochaska and Di Clemente’s theory and Miller and Rollnick’s model of motivational interviewing. Therapists first present the model of change with its different stages: pre-contemplation, contemplation, preparation, action, maintenance, and relapse. Patients and therapists then perform a decisional balance of four cases: the pros and cons of existing behavior and behavior change (the change considered can be abstinence or use reduction).
3	**Relatives** ● Inform about addiction ● Work on attitudes	Each patient can invite a relative (friend, family, or partner). The purposes are both to answer the relative’s questions about addiction and to aid the relative in helping the patient. The therapists give general information without disclosing personal and confidential information to the patient’s relative. The importance of confidentiality is stressed to patients and relatives.
4	**Coping with craving** ● Triggers list ● Craving definition ● How to cope with craving	This session starts with a brainstorming of what could lead to craving (situation, paraphernalia, propositions, etc.). The therapists then illustrates the craving curve, showing craving rising after triggers and cues; craving reduction after use; and even without drug use, showing the interest of surfing on the craving wave. The last part of this session is dedicated to finding solutions to cope with craving without using drugs.
5	**Decline a proposal** ● Define assertiveness ● How to refuse ● Role playing	The aim of this session is to learn how to decline to use/buy drugs through the assertiveness principle. Therapists start by defining assertiveness as standing up for your personal rights by expressing thoughts, feelings, and beliefs in direct, honest, and appropriate ways. Patients are trained to refuse, without aggressiveness and while taking body language into account, according to the following steps: Respond with a clear and firm “no”; Explain why you say no shortly and without justifying yourself; Broken record: Don’t add further explanations; Terminate the conversation. The final part of this session is role-playing where two patients act out a scene where one is a tempter and the other a user who has to refuse. The other patients are observers who note verbal and non-verbal assertive communication. A debriefing review what was well done and what needed to be improved. All patients perform both roles (tempter and tempted).
6	**Solving problems** ● Choose a patient’s problem ● Experiment the solving problem strategy	This session focuses on solving-problem techniques starting with a situation proposed by one participant. The steps are as follows: Define the problem, list all the possible solutions, for each solution list the pros and cons, pick the solution with the most pros and the fewest cons, evaluate the necessary means, note if those means are available, and if so, determine concretely how to implement the solution. The aim is not only to help one patient, but also to explain the technique to all patients.
7	**Expert patient (explain his drug use and care trajectory)**	A former patient (expert patient) who had completed the program in 2015 came to explain his addiction care path to other patients. He explained what steps he went through and what helped him at each step. Patients could intervene at any time to comment or ask questions.
8	**Invest time and money without drug** ● In a short/middle/long term perspective ● Develop planning strategies	In this session patients imagine what would be their life if that they had stopped using drugs. More time and more money will be available, both of which are triggers that could lead to craving. The therapists thus encourage patients to develop plans to invest their time and money in drug-free behaviors. Furthermore, the therapists suggest that those plans should be short, mid, and long-term without drugs. For the short term, it could be planning of what to do in the next few weeks.
9	**Content review** ● Open criticize group content ● Jacobson relaxation	The patients fill all the initial questionnaires again. A complete feedback of the group is collected. Patients are invited to criticize the organization and content of the sessions. Jacobson relaxation is then performed and patients can record it on their smartphones or receive it by e-mail.

### Statistical analysis

Variables are described using means (Standard Deviation) and percentages. When a patient did not answer all questionnaires, only available data was analyzed. This was also the case for drop outs. The distribution normality was checked. Comparison between pre and post intervention were tested using Khi^2^ and Repeated Measure Anova or Wilcoxon as appropriate, with a *p* < 0.05 threshold. The analysis were done using JASP 0.8.6.0 software.

## Results

### Population description

The 41 patients who came to at least one session were recruited between June 2017 and November 2019. They were on average 43 years old; 73% were men, 26% did not have their own housing, 58% had a job, and 58% were single. Regarding stimulant use, patients preferentially used cocaine (65%) over other stimulants and preferred snorting cocaine (68% vs. injection or smoking). The mean age at onset of stimulant use was 28 years old (± 10 years). They had an average of 1.2 grams or 3 rocks per day of stimulant use.

Concerning other substances, 61% were currently dependent on tobacco, 62% on alcohol, 12% on cannabis, and 2% on opiates (among whom all were on agonist maintenance treatment).

Regarding their current psychiatric component (according to their medical records), 40% did not have any psychiatric comorbidity, 30% had mood disorder, 12.5% personality disorder, 10% anxiety disorder, and 2% schizophrenia. Twenty nine percent of the patients had no prescribed psychotropic treatment, 33% had antidepressants, 37% antipsychotics [including aripiprazole prescribed as anti-craving treatment (46)], 28% benzodiazepines, and 28% mood-stabilizer treatment.

### Results and time course of the scores on assessment tools

Detailed results are presented in Table 2. The TLFB questionnaire showed that in the month before the group started, patients had a mean of 7 days of stimulant use. The median of the frequency of the number of days of abstinence was 76%. The OCCS craving mean total score was 18.9 (±7.4) in a range from 0 to 56, where a higher score indicates higher craving. The subscales were largely equivalent, with a mean obsessive score of 8.5 and a mean compulsive score of 10.4. The mean URICA score was 88 (±11.4) on a possible range of −16–112, where higher scores indicate greater motivation to change. The score can also be observed using stages. In this configuration, 25 patients had no equality between two stages and can be interpreted. We observe among those 25 patients that a half (52%) were in contemplation, 32% in action and 16% in maintenance; none was in pre-contemplation. The HAD anxiety mean score was 11.9 (±4.2) and the depression mean score was 7.9 (±4.7). Both scores highlight the presence of anxiety and depression symptoms (47). Furthermore, almost half of the population (*N* = 17), experiment depressive symptoms above the recommended screening cut off. Regarding anxiety screening, 90% of the participants experience symptoms above the cut off. The BSCQ self-confidence median was 350, the mean total score was 362 (±144) and the average score was 45.2 (±18.0), meaning that in the situations listed, patients felt that they were at a 45% risk of using the stimulant.

**TABLE 2:** Main assessment tools results.

	Pre-test *N* = 41			Post-test *N* = 17			Statistical pre-post tests
			
	Mean (SD)	Median	Frequency	Mean (SD)	Median	Frequency	Repeated measure ANOVA
TLFB (percentage of abstinence days in the previous month)	75.8 (27.5)	87.1		81.8 (23.2)	93.3		*p* = 0.268
OCCS total	18.9 (7.4)	18		16.5 (8.2)	17		*P* = 0.169
OCCS obsession	8.5 (3.9)	8		8.1 (3.9)	7		*P* = 0.858
OCCS compulsion	10.4 (4.3)	10		8.4 (4.8)	9		*P* = 0.083
URICA score	87.7 (11.4)	88		89.7 (10.4)	92		*P* = 0.870
URICA stages			0% pre- contemplation 52% contemplation 32% action 16% maintenance			0% pre- contemplation 54% contemplation 27% action 18% maintenance	
HAD depression	7.9 (4.7)	8	53.1% > cut off	6.6 (4.1)	6	28% > cut off	*P* = 0.556
HAD anxiety	11.9 (4.2)	11	91% > cut off	10.6 (3.5)	11	85% > cut off	*P* = 0.049
BSCQ total	361.9 (144.1)	350		444.7 (165.1)	496		*P* = 0.835
BSCQ mean	45.2 (18.0)	43.7		55.6 (20.6)	62		*P* = 0.833

SD, standard deviation; TLFB, timeline followback; OCCS, obsessive compulsive craving scale; URICA, university of rhodes island change assessment; HAD, hospital anxiety depression; BSCQ, brief self confidence questionnaire.

Among completers (*N* = 17), the percentage of abstinence days prior to the inclusion was 85.3% (measured with the TLFB) with no statistical diminution between baseline and the end of the intervention. There was no significant change either for the following variables: OCCS total score (mean 18.82 ± 7.6), obsession score (mean 8.2 ± 3.6), compulsion score (mean 10.4 ± 4.5); the BSCQ total score (mean 427.1 ± 115.5) or mean score (mean 53.4 ± 14.4); the HAD depression score (mean 7.1 ± 4.2) or the Anxiety score (12.1 ± 4.5), see Table 3. Among patients attending the last session, they did not all answer all questionnaires proposed. Furthermore, some tools were added after the beginning of the intervention, so were not proposed for the first patients.

**TABLE 3:** Completers evaluation.

	Pre-test *N* = 17			Post-test *N* = 17		
		
	Mean (SD)	Median	Frequency	Mean (SD)	Median	Frequency
TLFB (percentage of abstinence days in the previous month)	85.3 (17.1)	93.4		81.8 (23.2)	93.3	
OCCS total	18.8 (7.6)	19		16.5 (8.2)	17	
OCCS obsession	8.2 (3.6)	8		8.1 (3.9)	7	
OCCS compulsion	10.4 (4.5)	10		8.4 (4.8)	9	
URICA score	89.3 (12.9)	93		89.7 (10.4)	92	
URICA stages			0% pre-contemplation 60% contemplation 10% action 30% maintenance			0% pre-contemplation 54% contemplation 27% action 18% maintenance
HAD depression	7.1 (4.2)	9	53.8% > cut-off	6.6 (4.1)	6	28% > cut-off
HAD anxiety	12.1 (4.5)	12	84.6% > cut-off	10.6 (3.5)	11	85% > cut-off
BSCQ total	427.1 (115.5)	406		444.7 (165.1)	496	
BSCQ mean	53.4 (14.4)	50.6		55.6 (20.6)	62	

SD, standard deviation; TLFB, timeline follow back; OCCS, obsessive compulsive craving scale; URICA, university of rhodes island change assessment; HAD, hospital anxiety depression; BSCQ, brief self-confidence questionnaire.

### Number of sessions and attendance, patients’ views of the group session efficacy

**FIGURE 1:**
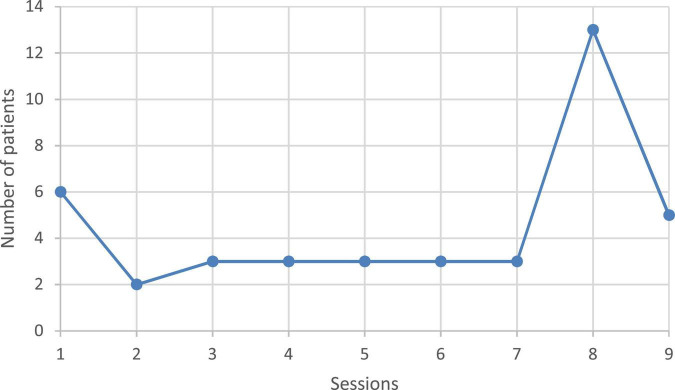
Attendance (number of sessions attended for each patient).

The average number of sessions attended by patients was 5.7 (±2.8) out of 9 sessions. The median was 7 and the mode was 8 sessions (see Figure 1). Figure 1 also highlight that 6 patients only attend one session and 5 went for all sessions. The TLFB did not significantly change between the beginning and the end of the intervention, the mean rate of abstinence days was 76% at the beginning and 82% at the end (see Table 2). Regarding the median, it was 87% at the beginning and at the end 60% of the population had scores higher than the initial median. Among the 41 patients who came to at least one session, 5 came to all sessions and 4/15 were abstinent at the end of the group, that is, 26% (among 15 patients) or 9% (among 41 patients). Among the 16 patients who attended the last session, on the URICA scale, 11 subjects described a specific stage. A half (54%) were in contemplation, 27% in action and 18% in maintenance. Those scores are very similar to those at the beginning of the intervention. About the HAD, among the 14 patients who completed the evaluation at the end of the group, 28% experience depressive symptoms above the cut off and 79% for anxiety. Those frequencies are lower than at the beginning of the intervention. Among pre-post evaluated patients (*N* = 13), a Khi^2^ was performed and depression rates above the cut-off significantly decrease (*p* = 0.026), this result is no longer significant for anxiety (*p* = 0.140).

Concerning self-efficacy, at the end of the intervention, 69% of the patients had a score above the median of the beginning of the group, what could suggest that they improve their self-efficacy feeling during the intervention, even if the attrition rate was high.

On the craving scale, 59% of participants diminish their total score on the OCCS between the beginning and the end of the intervention. This rate was lower on the obsession scale (41%) and higher on the compulsion scale (65%).

Participants’ feedback was positive, as per the comments of patients attending the last session. For example, verbatim included that some patients learnt theoretical elements and appreciate to be with peers. On the other hand, patients often told us that sessions could raise stimulant craving by talking about drugs.

## Discussion

We designed a specific relapse prevention CBT group for outpatients with stimulant use disorder to address their specific need (poly dependence, induced delusions, alteration in social and sexual behavior, neurocognitive impairment, and childhood trauma).

### Principal results

It is noteworthy that patients who attended the group sessions had 76% abstinent days in the month before the first session, raising the question of whether patients who came to the group were already highly motivated or had already initiated a change, such that only “*almost cured”* patients would attend this highly demanding type of care.

The observed OCCS total score was correlated with the frequency and intensity of use, and as patients had numerous abstinent days, their scores tended to be intermediate between high and low craving (5).

The URICA score is high (mean = 88), indicating that the patients who could attend the sessions had a high motivation to change (48).

Attendance could be interpreted in different ways. Attendance could be seen as quite high, with a mode of 8 out of 9 sessions. Looked at another way, we also note that only 12% of patients went to all sessions. Furthermore, compared to previous studies, the attendance was good. One study (24) reported that 26% of patients never came to any session, whereas for ours the rate was 9.7%, but they had 62% non-completers (completers were defined as patients who came to at least 6 consecutive sessions with cocaine-negative urine samples), whereas in this study 58% came to at least 6 sessions. Another study (a CBT and motivational open group program of 12 sessions) among 19 patients, reported a high retention rate (84%), defined as attending 11/12 sessions (25). In comparison, in our study the retention rate was 44%, defined as attending eight to nine sessions, among patients who came to at least one session.

We did not observe a significant improvement in cocaine use or questionnaires’ scores, but patients already had low scores at the beginning of the program. However, 4 of the 15 patients were abstinent at the end of the program.

### Limitations

However, this study has some limitations. This study is an open study with no other group (control or other intervention). We thus might lack the power to demonstrate significant differences or patient improvement. Some of this lack of power could be due to the use of self-rated questionnaires only.

This group is hard to organize, in part because practitioners in our clinic did not easily refer patients to group therapies, and in part because it is difficult to constitute a homogenous group. However, patients attending the last session (who were asked to comment on the group) gave positive feedback about the help provided by the group, the organization, and session content.

In the future, we would like to raise the effectiveness of the program, explore the differences between the different stimulants, and change the tools to gain in sensibility.

### Strengths

There are several strengths of this study. A specific intervention was designed to respond to the specific needs of stimulant user patients in a prospective study with pre-post evaluations. The study was conducted among patients affected by a disorders associated with poor compliance, and a good feasibility and acceptability were demonstrated. Moreover, this article partially responds to a previous article with a real life application of a group therapy in an out-patient treatment setting (49).

### Perspectives

To improve this study, it would be interesting to increase inclusions to demonstrate patient improvement. An *a priori* test (α = 0.05) for the difference between two means with matched pairs using the OCCS total score (one-tailed) suggests that the number needed to ensure sufficient statistical power is 67 patients, to whom both pre and post-evaluations would be applied. In order to confirm the efficacy, a comparison to another intervention, such as a computer-delivered program (50), is warranted.

Clinically, the content of the sessions could be enriched with mindfulness components and integrating psychoeducation on harm reduction on sexual behavior (51, 52).

This group intervention is feasible for patients suffering from stimulant use disorder and should be generalized in all care settings because the number of those patients is increasing in all care services without efficient pharmaceutical response.

## Conclusion

This study presents a specific targeted CBT group program for severe poly dependent patients suffering from stimulant use disorder. Indeed, few interventions exist for this specific population. This group program proved feasible, even if most patients had difficulties attending all sessions.

Patients’ recruitment in this study should go on to verify the efficacy of this therapeutic intervention. Furthermore, it would be interesting to add a follow up session, as well as to keep in contact with patients and to assess them again after the intervention.

## Data availability statement

The raw data supporting the conclusions of this article will be made available by the authors, without undue reservation.

## Ethics statement

Ethical review and approval was not required for the study on human participants in accordance with the local legislation and institutional requirements. Written informed consent for participation was not required for this study in accordance with the national legislation and the institutional requirements.

## Author contributions

EK and MF designed the group program. EK, KP, and NT included the participants and performed the intervention. LR and FV designed the research protocol. All authors contributed to manuscript revision, read, and approved the submitted version.
